# Temporal Relationship between HbA1c and Depressive Symptom Trajectories in a Longitudinal Cohort Study: The Mediating Role of Healthy Lifestyles

**DOI:** 10.3390/brainsci14080780

**Published:** 2024-07-31

**Authors:** Na Zeng, Chao Li, Huan Mei, Shuilin Wu, Chang Liu, Xiaokun Wang, Yanping Bao

**Affiliations:** 1 School of Public Health, Peking University, Beijing 100191, China; zengna19@bjmu.edu.cn (N.Z.); meihuan@pku.edu.cn (H.M.); 2211210073@stu.pku.edu.cn (S.W.); liuchang1@stu.pku.edu.cn (C.L.); xiaokun8527141@163.com (X.W.); 2 National Institute on Drug Dependence and Beijing Key Laboratory of Drug Dependence, Peking University, Beijing 100191, China; 3 Department of Anesthesiology, Beijing Friendship Hospital, Capital Medical University, Beijing 100050, China; lichao2988@mail.ccmu.edu.cn

**Keywords:** HbA1c, depressive symptoms, healthy lifestyle, cross-lagged panel model, trajectories, mediating effect

## Abstract

This study analyzed China Health and Retirement Longitudinal Study data to explore the HbA1c–depression link, including depressive trajectories, while considering the mitigating impact of healthy lifestyles. Cross-lagged panel models and group-based trajectory modeling were performed to investigate the temporal relationship between HbA1c levels and depressive symptoms, as well as the depressive trajectories. Structural equation models were used to assess the mediating effects of healthy lifestyles. The mean age of the participants was 57.66 ± 9.04 years, with 53.68% being female. Analyzing 8826 participants across three waves, we observed a significant prediction of subsequent depressive symptoms by the preceding HbA1c levels (β = 0.296; *p* < 0.001). Four distinct trajectories of depressive symptoms were distinguished: stable low, stable moderate, increasing, and stable high. Elevated HbA1c levels were associated with a higher risk of developing stable high (OR 1.12 and 95% CI 1.02–1.23), increasing (OR 1.21 and 95% CI 1.11–1.32), and stable moderate depressive symptoms (OR 1.07 and 95% CI 1.01–1.13). Engaging in two healthy life behaviors reduced stable high and increasing depressive pattern risks by 32% and 30%, respectively. Adherence to a healthy lifestyle lessened 7.2% of the impact of high HbA1c levels on the subsequent depressive symptoms. These findings highlight the potential benefits of incorporating adequate sleep and light physical activities, which might reduce the adverse impact of elevated HbA1c levels on depressive symptoms.

## 1. Introduction

It is well established that type 2 diabetes (T2DM) and depression have often co-existed. Among patients with T2DM, the prevalence rate of depression ranged from 10.3% to 37.9% [1,2,3,4,5,6,7]. Individuals with type 2 diabetes have a 40–60% increased risk of developing depression [8]. The comorbidity of depression and T2DM poses significant challenges, such as poor disease management [9], decreased quality of life [10], higher healthcare expenditure [11], and increased mortality [12]. Nevertheless, the precise nature of this relationship remains elusive. Glycated hemoglobin (HbA1c) serves as the gold standard for assessing glycemic control in patients with diabetes mellitus, reflecting glucose exposure over the previous two to three months. Previous studies have identified an association between HbA1c levels and depressive symptoms [1,13]; however, the correlation between HbA1c levels and depression has yielded mixed findings in the literature. Three studies found that higher HbA1c levels were associated with an increased risk for subsequent depression [14,15,16], and four reported no significant results [17,18,19,20]. Most evidence supporting these associations relies on a single measure of depressive symptoms. However, given the fluctuating and recurring nature of depressive symptoms, relying solely on a single measure could distort the true relationship between HbA1c and depressive status. Although one study explored a potential link between HbA1c and the trajectory of depressive symptoms, it failed to establish a temporal sequence [15].

Temporal sequential associations between diabetes and depression carry important implications for the clinical care and treatment of both conditions. Healthy lifestyle factors, such as physical activity and sleep duration, may influence mental well-being and the risk of diabetes in later life [21,22,23,24,25]. Studies found that small doses of moderate-to-vigorous physical activity could be beneficial to both depression and cardiometabolic health [21,25]. A meta-analysis indicated that normal sleep duration might decrease the risk of depression by 30–40% [26], and insomnia treatment can improve the depressive symptoms [27]. Additionally, the amount and quality of sleep is associated with changes in energy homeostasis and insulin resistance [28,29,30], and specifically, frequent insomnia could cause higher HbA1c levels [31]. However, research on the potential mediating role of healthy lifestyles in the association between glycemic control and depression, as well as quantifying the extent to which healthy lifestyles may alleviate this relationship, is limited. Understanding the relationship between these conditions and the mediating effects of healthy lifestyles would enable clinicians to customize their approach to caring for patients and potentially lead to improved prevention and management strategies with these conditions.

Therefore, this study aims to investigate the temporal relationship between HbA1c levels and depressive symptoms using a cross-lagged panel design. We will further explore how baseline HbA1c relates to longitudinal patterns of depressive symptom trajectories, after excluding high-risk participants at baseline. Structural equation modeling (SEM) will be employed to assess whether healthy lifestyle behaviors, such as light physical activity and adequate sleep, can mitigate the impact of high HbA1c levels on elevated depressive symptoms. We hypothesize that there is a temporal sequence between baseline HbA1c levels and subsequent depressive symptoms, particularly in individuals with increasing and consistently high levels of depressive symptoms. Additionally, we posit that healthy lifestyles may mediate this association.

## 2. Methods

### 2.1. Participants

The data were obtained from the China Health and Retirement Longitudinal Study (CHARLS), a nationally representative prospective cohort survey targeting adults aged 45 years and above. Detailed information regarding CHARLS’s study design and sampling methodologies has been previously documented (the detailed information regarding the study can be found at https://charls.charlsdata.com/pages/data/111/en.html, accessed on 12 April 2024) [32]. In brief, the baseline survey employed a multistage, stratified probability-proportionate-to-size sampling approach, enrolling 17,708 adults from 450 villages or urban communities spanning 150 counties across 28 provinces in China. The baseline survey was initiated in 2011 (wave 1), with subsequent waves completed approximately every 2 years, and data from wave 2 and wave 3 were collected in 2013 and 2015, respectively. This study included participants aged 45 years or older at baseline, with data available for assessing depressive symptoms and healthy lifestyles in waves 1, 2, and 3 and HbA1c in wave 3. To investigate the longitudinal association between HbA1c and depressive symptoms, we excluded those who scored higher than 20 on the CES-D-10 at baseline from the depressive trajectories analysis.

**Figure 1:**
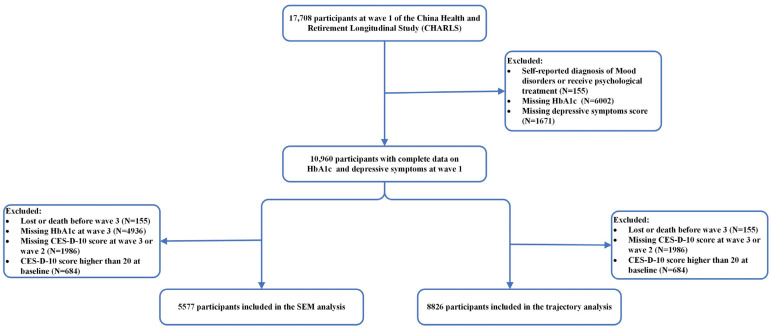
Flow chart of the participant selection.

A total of 17,708 participants were initially enrolled in CHARLS 2011. Participants with diagnosed psychiatric disorders at baseline (*n* = 155), lacking HbA1c data at baseline (*n* = 6002), or without an assessment of depressive symptoms at baseline (*n* = 1671) were excluded at first. Further, to examine longitudinal associations, participants were additionally excluded due to loss or death before wave 3 (*n* = 155) or not completing the blood test at wave 3 (*n* = 4936) or missing the depressive symptoms assessment (*n* = 1482 at wave 2 and 524 at wave 3) or scored CES-D-10 higher than 20 at baseline (*n* = 684). Consequently, the final analytic sample comprised 5577 participants for the cross-lagged panel model analysis and 8826 participants for the longitudinal trajectories analysis (Figure 1).

### 2.2. Blood Sample Collection and Measurements of HbA1c

The participants in the CHARLS 2011 and 2015 waves were required to fast overnight before having their blood samples drawn by personnel with medical training. The samples were then promptly transported to the nearby laboratory, where they were stored at 4 °C. Prior to analysis, the blood samples were transported to the central laboratory in Beijing, where they were frozen at −80 °C, after having been centrifuged and stored at −20 °C. The HbA1c levels in the venous blood samples were determined by boronated affinity liquid chromatography following an overnight fasting period.

### 2.3. Assessment of Depressive Symptoms

The assessment of depressive symptoms in this study was performed utilizing the Center for Epidemiologic Studies Depression Scale (CES-D-10), which consists of ten items. The participants were asked how often they had experienced any of the ten symptoms listed during the last week (e.g., felt depressed, felt sad, sleep was restless, felt lonely, bothered by little things, everything was an effort, was happy, enjoyed life, had trouble keeping in mind what is going on, and could not get going). The answers to the 10 items were categorized as follows: rarely or never (<1 day), sometimes (1–2 days), occasionally (3–4 days), and most or all of the time (5–7 days). Each item was assigned a score from 0 (rarely) to 3 (most or all of the time). Before summing up the scores, items 5 and 8 were reverse-scored. The total score for the CES-D-10 ranged from 0 to 30. Based on prior research, individuals who obtained a total score of 20 or above were categorized as depressed [33,34]. The CES-D-10 has undergone comprehensive validation for application in general populations and has demonstrated adequate reliability and validity for use with community-dwelling older adults in China [35].

### 2.4. Definition of Healthy Lifestyle

We considered 2 lifestyle factors, including light physical activity and sleep duration, according to the former studies that could modify depressive symptoms in the Chinese population [36,37], and the score for each factor was categorized as yes (score of 1) or no (score of 0). Healthy physical activity was defined as engaging in walking solely for recreation, sport, exercise, or leisure, with a frequency of no less than 3 times per week and a duration of at least 30 min each time. If the participants engaged in healthy physical activity, then the score for physical activity was considered as 1; otherwise, it was 0. For sleep duration, if the participants reported sleep 7–8 h on a normal night, the score for sleep duration was regarded as 1; otherwise, it was 0. We calculated an overall score of modifiable lifestyle factors based on these two components. The score ranged from 0 to 2, with higher scores indicating greater adherence to a healthy lifestyle. Then, we categorized the participants into two distinct groups of modifiable risk: those with a healthy lifestyle received a score of 2, while those with an unhealthy lifestyle received a score ranging from 0 to 1.

### 2.5. Covariates

The covariates were selected based on previous epidemiological evidence and data availability at baseline [15,38,39]. The potential confounders included age, sex (male and female), BMI (<25 kg/m^2^ and ≥25 kg/m^2^), residential area (urban and rural), education level (illiterate, primary school, middle school, high school, and college or above), average household income (<1000, 1000, 5000, 10,000, and 20,000 CNY), marital status (married, separated, and unmarried/divorced/widowed), smoking status (never, current, and previous), alcohol drinking (never, current, and previous), and presence of comorbidity with physical diseases. Cognitive function was assessed by the Mini-Mental State Examination (MMSE) results [40].

### 2.6. Statistical Analysis

Categorical variables are represented as numbers (proportions), whereas continuous variables are represented as the means and standard deviations (SDs) or median and interquartile range (IQR) based on the data distribution.

**Figure 2:**
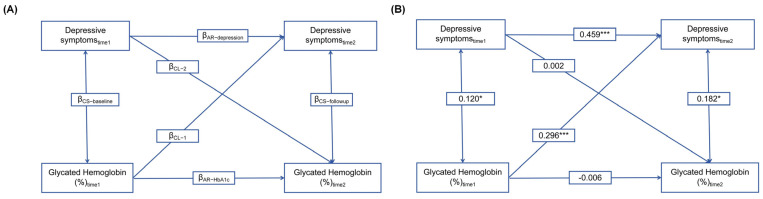
The cross-lagged panel models applied to assess the HbA1c levels and depressive symptoms among Chinese adults. (**A**) The cross-lagged theoretical regression models for the temporal relationship between depressive symptoms and HbA1c levels; (**B**) the cross-lagged panel model where the HbA1c levels at wave 1 were associated with the depressive symptoms at wave 3. * *p* < 0.05 and *** *p* < 0.001.

In order to investigate the potential longitudinal relationship between HbA1c levels and CES-D-10 scores, cross-lagged path models were performed at 2 time points (wave 1 and wave3), 4 years apart. The general modeling strategy of this study is illustrated in Figure 2A, including 6 paths and their corresponding coefficients. To begin with, cross-lagged paths consist of two cross-lagged coefficients, denoted as β_CL-1_ and β_CL-2_. β_CL-1_ represents the effect of HbA1c at time 1 on the CES-D-10 scores at time 2, while β_CL-2_ implies the reversal of the relationship between the CES-D-10 scores at time 1 and the HbA1c levels at time 2. The temporal sequence was investigated through the evaluation of the estimated standardized cross-lagged coefficients [41]. Subsequently, the cross-sectional relationships between the HbA1c levels and cognitive scores were further examined. The coefficient β_CS-Baseline_ at time 1 signified the baseline correlation between the HbA1c levels and CES-D-10 scores, whereas the coefficient β_CS-follow-up_ at time 2 denoted the correlation between the HbA1c levels and CES-D-10 scores at time 2. Finally, 2 autoregressive coefficients, denoted as β_AR-HbA1c_ and β_AR-depression_, were derived from the path-connecting times 1 and 2 for HbA1c and the CES-D-10 scores, respectively. These coefficients accounted for the stability of the individual in each measure from the time of the baseline to the subsequent time. Before the cross-lagged path analysis, the HbA1c levels and scores were normalized using Z-transformation (mean 0 and SD 1) and then they were adjusted for a series of covariates by regression residual analyses (including age, sex, residence, marital status, educational level, smoking and drinking status, number of comorbid physical diseases, cognitive scores, BMI, and healthy life score).

Moreover, to explore the relationship between the HbA1c levels at wave 1 and the longitudinal trajectories in the depressive symptoms from 2011 to 2015, a latent mixed model analysis was conducted [42]. First, 5 types of trajectories were established and subsequently compared with models featuring 4, 3, 2, and 1 trajectories using the Bayesian information criterion (BIC) to evaluate the goodness of fit for each model. Then, models with varying functional forms were evaluated by scrutinizing the significance levels of cubic, quadratic, and linear terms. Finally, our model comprised two classes with linear-order terms and two classes with quadratic-order terms. The mean posterior estimated probability of the final group membership was utilized to assess discrimination. To investigate the relationship between the HbA1c levels and depressive symptom trajectories, we performed multinomial logistic regression on the latent trajectory subgroups obtained from the latent mixed models. We adjusted for the covariates as cross-lagged panel models. Moreover, a subgroup analysis was performed to investigate whether the association between HbA1c and the depressive symptom trajectories varied by age, sex, and healthy lifestyle.

In the third step, we assessed the potential mediating effect of a healthy lifestyle during wave 3 between the HbA1c levels in wave 1 and the CES-D-10 scores in wave 3. A structural equation model (SEM) was utilized to estimate the standard path coefficients and evaluate the direct association between the HbA1c levels at wave 1 and the CES-D-10 scores at wave 3, along with the indirect associations mediated through a healthy lifestyle at wave 2. In the mediation analysis, the bootstrap method was employed with 1000 samples. A significant mediating effect was indicated if the 95% confidence interval (CI) did not include zero. A comparative fit index (CFI) > 0.90, standardized root mean square residual (SRMR) < 0.05, Tucker–Lewis Index (TLI) > 0.90, root mean square error of approximation (RMSEA) < 0.05, and goodness-of-fit index (GFI) > 0.90 were used to suggest the good fitness of the cross-lagged panel models and SEM models [43]. All the tests were two-tailed, and *p* values of less than 0.05 were considered statistically significant. All the analyses were conducted using SAS software Version 9.4 and R version 4.0.2

## 3. Results

### 3.1. Association of HbA1c Levels and Depressive Symptoms by Cross-Lagged Analysis

Ultimately, a national sample of 5577 adults aged 45 years or older were included in the cross-lagged analysis, with approximately half of them being female (2994/5577, 53.68%). The mean age of the participants at baseline was 57.66 (SD 9.04) years (Table 1). The baseline characteristics of the participants regarding HbA1C are presented in Table 1. The overall mean (±SD) HbA1C of the study population was 5.23 ± 0.75%. Among the participants, 5344 (95.82%) had normal HbA1C levels, while 79 (1.42%) had poorly controlled HbA1C levels exceeding 8%. Further, 333 (5.97%) participants reported having been diagnosed with diabetes by doctors, with 182 (3.26%) taking medicine for treatment.

Figure 2B illustrates the cross-lagged model exploring the temporal relationship between the HbA1c levels and depressive symptoms. After controlling for covariates, the cross-lagged model fitted the data adequately (CFI = 0.999; SRMR = 0.002; RMSEA = 0.001; TLI = 0.999; and GFI = 0.997). Depressive symptoms at the time 1 point were positively related to themselves over time (β = 0.459; *p* < 0.001), which indicated a relatively strong autoregressive effect for depressive symptoms over 4 years. Significant cross-lagged effects were observed, indicating that the preceding HbA1c levels significantly predicted depressive symptoms at time 2 (β = 0.296; *p* < 0.001), while the reverse relationship was not significant, which means that depressive symptoms did not predict HbA1c levels at T2 (β = 0.002; *p* = 0.329).

Figure S1 shows the subgroup analysis of the cross-lagged models by sex, age, and adherence to a healthy lifestyle. The higher levels of HbA1c significantly associated with subsequent depressive symptoms were generally observed across a middle-aged, female, and healthy and unhealthy population, while they were not significant among old and male participants. All of the models fit well (CFI > 0.95; SRMR < 0.05).

### 3.2. Predictive Relationship between HbA1c Levels and Longitudinal Trajectories of Depressive Symptoms, and the Ameliorating Impact of Healthy Lifestyles

**Figure 3:**
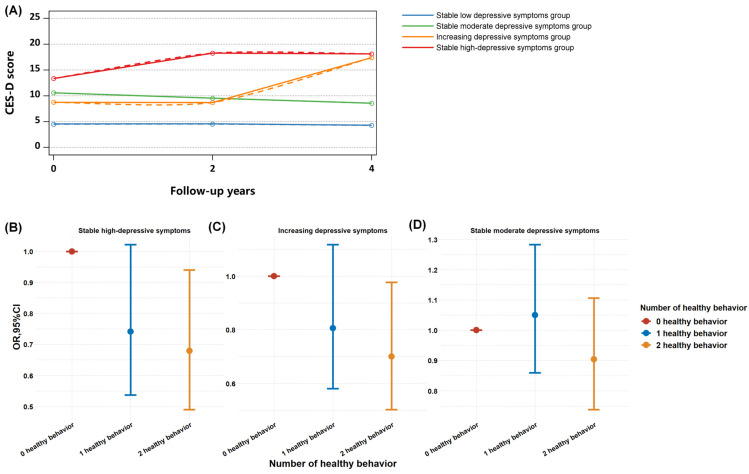
The depressive symptom trajectories during 2011 to 2015 and the mitigated effect of healthy life behavior on the risk of the depressive trajectories group; (**A**) the trajectories of the depressive symptoms (the dashed lines represent the 95% CI of the trajectories); (**B**) healthy lifestyle on the risk of stable high depressive symptoms; (**C**) healthy lifestyle on the risk of increasing depressive symptoms; and (**D**) healthy lifestyle on the risk of stable moderate depressive symptoms. The mediating role of a healthy lifestyle between the HbA1c levels and depressive symptoms.

Four discrete trajectories in depressive symptoms during the 4-year span were identified (Figure 3): 4809 participants (54.5%) maintained a low level of depressive symptoms throughout (low stable depressive group (LSD group); mean range, CES-D-10 median score 6.0 to 6.0); 2759 (31.3%) had stable moderate depressive symptoms throughout (stable moderate depressive group (SMD group); CES-D-10 median range, 7.0 to 9.0); 616 (7.0%) started with normal levels and experienced an increase in depressive symptom scores (increasing depressive symptoms group (ID group); mean depressive symptoms increase, 7.0 to 10.0); and 642 (7.2%) maintained a high level of depressive symptoms (stable high depressive symptoms group (SHD group); median CES-D-10 scores, 10.5 to 11.0). The mean (SD) probabilities for each individual being in the final group were 0.87 (0.15), 0.75 (0.15), 0.72 (0.18), and 0.81 (0.18) across the trajectory groups. The basic characteristics of the participants at baseline by the depressive symptom trajectories are shown in Table 2. Compared to those with low depressive symptoms, individuals with higher or increasing depressive symptoms were more likely to be older, female, living in a rural area, and unmarried.

Further, we explored the association between the HbA1c levels and different depressive symptom trajectories. In comparison to the stable low depressive symptoms group, the participants with elevated HbA1c levels were more likely to have stable high depressive symptoms (OR 1.12 and 95% CI 1.02–1.23), increasing depressive symptoms (OR 1.21 and 95% CI 1.11–1.32), and stable moderate depressive symptoms (OR 1.07 and 95% CI 1.01–1.13). In the subgroup analysis, the positive association between elevated HbA1c levels and stable high-level depressive symptoms was also observed in middle-aged adults (OR 1.17 and 95% CI 1.04–1.32) and participants not adhering to healthy lifestyles (OR 1.14 and 95% CI 1.01–1.30). A consistent significant positive association between elevated HbA1c levels and increasing depressive symptoms was found in all the subgroups except for male adults and participants adhering to healthy lifestyles. As for the stable moderate depressive symptoms group, significant increased HbA1c levels were found only in older participants (OR 1.1 and 95% CI 1–1.2) and male adults (OR 1.11 and 95% CI 1.02–1.21) (Table 3).

Then, the association between the number of healthy life behaviors was also compared between the four trajectory groups. The results showed that compared with zero healthy life behaviors, two healthy life behaviors could significantly reduce the risk of a high level of depressive symptoms (OR = 0.68 and 95% CI 0.49–0.94) and increasing depressive symptoms (OR = 0.70 and 95%CI 0.50–0.97) (Figure 3B–D).

**Figure 4:**
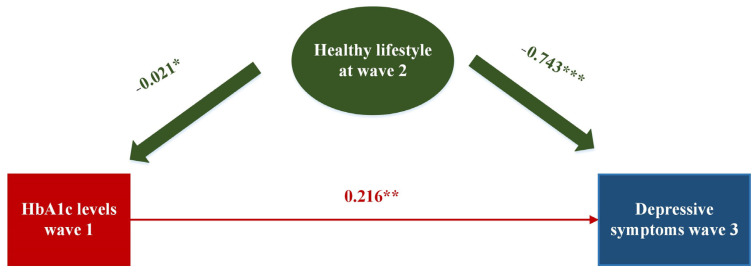
Healthy lifestyles at wave 2 as potential mediators between HbA1c levels at wave 1 and depressive symptoms at wave 3. Standardized regression coefficients are presented. All parameters were estimated simultaneously with a structural equation model. * *p* < 0.05, ** *p* < 0.01, and *** *p* < 0.001.

The results of the mediation analyses are presented in Figure 4. In our SEM analysis, we incorporated healthy lifestyle choices as potential mediators. Notably, our findings indicated that baseline levels of HbA1c significantly predicted subsequent depressive symptoms at follow-up (β = 0.216 and *p* = 0.006), after adjusting for covariates. An indirect association also suggested that the relationship between baseline HbA1c levels and depressive symptoms at follow-up is partially alleviated by healthy lifestyle-related behaviors (model fit: CFI = 0.982; RMSEA = 0.031; RMSEA = 0.015; TLI = 0.973; and GFI = 0.998). We observed that adherence to a healthy lifestyle at wave 2 was significantly associated with depressive symptoms at wave 3 (β = −0.743 and *p* < 0.001) and was associated with decreased HbA1c levels at wave 1 (β = −0.021 and *p* = 0.034) (see Figure 4), which could mitigate 7.2% of the association between the HbA1c levels and subsequent depressive symptoms. The significance of the mediation effect was tested using the Bootstrap program. As shown in Table 4, the 95% confidence interval of the mediation path did not include 0, indicating that the mediation effect was significant. In the subgroup analysis, healthy lifestyles were found to reduce the association between HbA1c and depressive symptoms by 7.9% in middle-aged participants and by 2.3% in female participants (Figure S2).

## 4. Discussion

This study provides novel insights into the temporal association between HbA1c levels not only for a single measure of subsequent depressive symptoms but also for sustained elevations in depressive symptoms over a longitudinal span of four years. In comparison to trajectories characterized by stable low levels of depressive symptoms, which is indicative of a healthy mood pattern, baseline HbA1c levels were observed to substantially increase the risk of trajectories marked by stable moderate, increasing, and high-level depressive symptoms. Moreover, participants adhering to two healthy lifestyle factors exhibited a significant reduction in the risk of experiencing increasing and high-level depressive symptom trajectories. Specifically, the incorporation of simple healthy lifestyle practices including adequate sleep and light physical activity during leisure time demonstrated a noteworthy ability to alleviate approximately 7.2% of the adverse impact that elevated HbA1c levels exerted on depressive symptoms.

Our findings were supported by an English population study, indicating a weak but significant temporal link between HbA1c levels and depressive symptoms [16]. In our study, there was no correlation between HbA1c at time 1 and HbA1c at time 2. This lack of correlation could be attributed to lifestyle changes, such as diet, physical activity, or medication adherence, which can drastically affect HbA1c levels [44,45]. Additionally, weight changes and diabetes education programs can also lead to variations in HbA1c over time [46,47], making it difficult to establish a direct correlation between HbA1c levels from two years ago and this year. To further validate our findings, we explored the association between baseline HbA1c levels and longitudinal patterns of depressive symptoms. Elevated baseline HbA1c levels exhibited a significant association with all three trajectories of elevated depressive symptoms compared to a healthy mood pattern. Particularly, they posed a notably higher risk in individuals exhibiting a stable high level of depressive symptoms and those showing an increasing trend of depressive symptoms over time. Fortunately, we found that combined healthy lifestyles involving simply sleep over 6 h and light physical activity at leisure time could mitigate about 32% of the risk of a stable high level of depressive symptoms and 30% of an increasing trend of depressive symptoms, respectively. Our findings are supported by a previous study that also identified four trajectories of depressive symptoms. This prior research highlighted that those individuals in the highest quintile of HbA1c (≥5.6%) exhibited an increased risk of developing increasing and high depressive symptoms compared to those in the lowest quintile (≤4.8%) [15]. Differing from the aforementioned study, we excluded participants with reported psychiatric disorders or CES-D-10 scores exceeding 20 at baseline, thereby establishing a cohort study population without depression at baseline, which allowed for a more comprehensive examination of the temporal relationship between HbA1c and depressive symptom trajectories. Moreover, we employed HbA1c as a continuous measure to investigate the linear contribution of HbA1c to the risk of depressive trajectories, enhancing our statistical power to detect differences.

Prior studies have indicated that individuals exhibiting elevated levels of HbA1c tend to have shorter durations of sleep and engage in less physical activity [25,38,48,49,50], which might increase the risk of developing depressive symptoms [21,22,23,51,52]. Our research indicated a dynamic interplay between HbA1c levels and depressive symptoms, with potential mitigating effects observed from a longer sleep duration and participation in light physical activity. Consistent with our study, an English study suggested lifestyle behaviors like smoking, diet, and physical activity could mediate this connection. However, these behaviors, in the study, were positively associated with both HbA1c levels and depressive symptoms, failing to reduce the impact of elevated HbA1c levels on depressive symptoms [16]. Another prior study showed that adherence to physical activity and glycemic control together reduced depression risk in females and the elderly. Nevertheless, evidence for glycemic control mediating the pathway from physical activity to depression was insignificant [24]. Additionally, a meta-analysis suggests that a normal sleep duration is associated with decreased HbA1c levels [29], and a Mendelian randomization study showed insomnia causes higher HbA1c levels [31]. Additionally, a randomized trial found that a lifestyle intervention, including light physical activity and improved sleep, was associated with lower HbA1C levels and a reduction in depressive symptoms [53]. Another study found physical exercise can enhance sleep quality in older adults with T2DM, reduce depression, and delay the onset of cognitive impairment [54]. Our findings carry significant implications for the implementation of lifestyle-based preventive interventions aimed at reducing the risk of depression among individuals at increased risk of diabetes. Merely depending on glycemic control may not work as a sufficient preventive measure for future depression. Concurrently, lifestyle-related behaviors such as ensuring sufficient sleep and participating in light physical activities should be considered essential components of preventive measures.

To our knowledge, this study was the first attempt to examine the temporal relationship between HbA1c levels, depressive symptoms, and the trajectories of depressive symptoms, as well as the potential mitigating effect of healthy lifestyles among Chinese middle-aged and older adults. Our study implemented the repeated measurement of HbA1c levels and depressive symptoms as well as the availability of healthy lifestyle variables in a large population-based sample of middle-aged and elderly people. We incorporated both a cross-lagged analysis and a structural equation modeling approach, enabling us to depict the dynamic relationship between HbA1c levels and depressive symptoms and the potential mediation effect of healthy lifestyles.

Some limitations should also be taken into consideration when interpreting our results. Firstly, the loss to follow-up poses a challenge in large cohorts of general populations, like CHARLS. There is a possibility that individuals who suffer with depressive symptoms may have an increased tendency to withdraw from the study, which could result in an underestimate of the true relationships between HbA1c levels and depression. Secondly, our analysis included an approximate 4-year follow-up interval, primarily due to the limited availability of blood samples at later wave marks. Consequently, it remains undetermined whether this association persists over an extended period. Thirdly, while the predictive associations between HbA1c levels and depressive symptoms were statistically significant after adjusting for a variety of potential confounding factors, there may still be additional related but uncontrolled variables at play, including inflammation factors (e.g., IL-6 and TNF-α), kidney failure (e.g., serum creatinine and blood urea nitrogen), and hemoglobinopathies. Regrettably, data on these variables are not available in the CHARLS dataset. Fourthly, considering the assessment of physical activity and sleep duration were self-reported data, participants may have difficulty in recalling accurately and might report what they perceive to be socially acceptable rather than their actual behaviors. To address these potential biases, future studies should incorporate wearable devices such as accelerometers, pedometers, and sleep monitors to validate self-reported data and reduce the reliance on participant recall. Finally, diets rich in fruits, vegetables, fish, and whole grains, which are known to support stable blood glucose levels and promote mental well-being, were not considered in the analysis due to a lack of data. This aspect should be taken into account in future studies. Additionally, future studies can conduct randomized controlled trials to validate the effectiveness of lifestyle-based interventions and explore the integration of psychological interventions, such as cognitive-behavioral therapy (CBT), with lifestyle modifications to determine their combined effect on reducing depressive symptoms in individuals at risk of diabetes.

## 5. Conclusions

Our study reveals a temporal relationship between HbA1c levels and depressive symptoms and their changing trajectories, suggesting potential mediation by healthy lifestyle behaviors. These findings emphasize the significance of incorporating simple yet effective lifestyle interventions into preventive strategies for individuals dealing with a comorbidity of diabetes and depression. By implementing such strategies, healthcare providers can enhance patient outcomes, improve overall mental well-being, and potentially reduce the incidence of severe depressive episodes among those with diabetes. This approach not only supports better glucose control but also promotes a holistic and comprehensive management plan for individuals facing the dual challenge of diabetes and depression.

## Figures and Tables

**Table 1:** The baseline characteristics of the study population (N = 5577).

Characteristic	Total
Age (years), (mean ± SD)	57.66 ± 9.04
Gender, N (%)	
Male	2583 (46.32)
Female	2994 (53.68)
Residential area, N (%)	
Urban	1815 (32.54)
Rural	3762 (67.46)
Education, N (%)	
Illiterate	1572 (28.19)
Primary school	2269 (40.68)
Middle school	1173 (21.03)
High school/vocational high school	505 (9.06)
Junior college or above	58 (1.04)
Average household income (CNY), N (%)	
<1000	2552 (45.76)
1000–5000	754 (13.52)
5000–10,000	404 (7.24)
10,000–20,000	523 (9.38)
>20,000	1344 (24.10)
Marital status, N (%)	
Married	4584 (82.19)
Separated	241 (4.32)
Unmarried/divorced/widowed	752 (13.48)
Ever/current smoke, N (%)	
No	3130 (56.12)
Yes	1500 (26.90)
Ever smoke	947 (16.98)
Ever/current alcohol, N (%)	
No	3050 (54.69)
Yes	1834 (32.89)
Ever alcohol	93 (12.43)
Daily sleep time (hours, mean ± SD)	6.35 ± 1.47
Physical comorbidities, N (%)	
No	1658 (30.79)
Yes	3727 (69.21)
MMSE score, (median, Q_25_–Q_75_)	15.00 (12.00, 17.50)
BMI (kg/m^2^, mean ± SD)	24.40 (33.61)
CES-D-10 score at wave 1 (median, Q_25_–Q_75_)	7.00 (4.00, 12.00)
CES-D-10 score at wave 2 (median, Q_25_–Q_75_)	7.00 (4.00, 11.00)
CES-D-10 score at wave 3 (median, Q_25_–Q_75_)	7.00 (3.00, 12.00)
Healthy lifestyle, N (%)	
0–1 healthy lifestyle	3178 (56.98)
2 healthy lifestyle	2399 (43.02)
With diabetes, N (%)	
No	5244 (94.03)
Yes	333 (5.97)
Taken medicine for diabetes, N (%)	182 (3.26)
HbA1C (mmol/L, mean ± SD)	5.23 ± 0.75
HbA1C levels	
<6.4% mmol/L	5344 (95.82)
6.4%~8.0% mmol/L	154 (2.76)
>8.0% mmol/L	79 (1.42)

**Table 2:** The characteristics of the participants in the different depressive trajectory groups at baseline.

Variables	LSD Group (N = 4809)	SMD Group (N = 2759)	ID Group (N = 616)	SHD Group (N = 642)	Total (N = 8826)	*p* Value *
Age (years), (mean ±SD)	57.76 ± 9.26	58.64 ± 9.51	59.01 ± 9.31	57.95 ± 8.71	58.14 ± 9.31	<0.001
Gender, N (%)						
Male	2578 (53.61)	1115 (40.41)	217 (35.23)	193 (30.06)	4103 (46.49)	<0.001
Female	2231 (46.39)	1644 (59.59)	399 (64.77)	449 (69.94)	4723 (53.51)	
Residential area, N (%)						
Urban	1979 (41.15)	895 (32.44)	154 (25.00)	175 (27.26)	3203 (36.29)	<0.001
Rural	2830 (58.85)	1864 (67.56)	462 (75.00)	467 (72.74)	5623 (63.71)	
Education, N (%)						
Illiterate	1081 (22.48)	826 (29.94)	226 (36.69)	243 (37.85)	2376 (26.92)	<0.001
Primary school	1871 (38.91)	1237 (44.84)	261 (42.37)	280 (43.61)	3649 (41.34)	
Middle school	1163 (24.18)	495 (17.94)	98 (15.91)	84 (13.08)	1840 (20.85)	
High school/vocational high school	610 (12.68)	181 (6.56)	30 (4.87)	34 (5.30)	855 (9.69)	
Junior college or above	84 (1.75)	20 (0.72)	1 (0.16)	1 (0.16)	106 (1.20)	
Average household income (CNY), N (%)						
<1000	2276 (47.33)	1259 (45.63)	260 (42.21)	264 (41.12)	4059 (45.99)	<0.001
1000–5000	501 (10.42)	412 (14.93)	81 (13.15)	104 (16.20)	1098 (12.44)	
5000–10,000	281 (5.84)	212 (7.68)	63 (10.23)	65 (10.12)	621 (7.04)	
10,000–20,000	400 (8.32)	267 (9.68)	59 (9.58)	79 (12.31)	805 (9.12)	
>20,000	1351 (28.09)	609 (22.07)	153 (24.84)	130 (20.25)	2243 (25.41)	
Marital status, N (%)						
Married	4110 (85.46)	2168 (78.58)	474 (76.95)	488 (76.01)	7240 (82.03)	<0.001
Separated	198 (4.12)	137 (4.97)	24 (3.90)	27 (4.21)	386 (4.37)	
Unmarried/divorced/widowed	501 (10.42)	454 (16.46)	118 (19.16)	127 (19.78)	1200 (13.60)	
Ever/current smoke, N (%)						
No	2499 (52.00)	1638 (59.41)	385 (62.50)	425 (66.20)	4947 (56.08)	<0.001
Yes	1420 (29.55)	680 (24.66)	140 (22.73)	129 (20.09)	2369 (26.86)	
Ever smoke	887 (18.46)	439 (15.92)	91 (14.77)	88 (13.71)	1505 (17.06)	
Ever/current alcohol, N (%)						
No	2484 (51.69)	1612 (58.47)	364 (59.09)	421 (65.58)	4881 (55.33)	<0.001
Yes	1775 (36.93)	800 (29.02)	170 (27.60)	141 (21.96)	2886 (32.72)	
Ever drink alcohol	547 (11.38)	345 (12.51)	82 (13.31)	80 (12.46)	1054 (11.95)	
Daily sleep time, (hours), (mean ± SD)	6.40 (1.54)	6.29 (1.60)	6.20 (1.59)	6.25 (1.61)	6.34 (1.57)	0.002
Physical comorbidities, N (%)						
No	25 (0.52)	23 (0.83)	2 (0.32)	6 (0.93)	56 (0.63)	0.205
Yes	331 (6.89)	288 (10.45)	64 (10.39)	97 (15.13)	780 (8.85)	<0.001
MMSE score, (median, Q_25_–Q_75_)	16.00 (13.00, 19.00)	14.00 (11.00, 17.00)	13.42 (10.00, 16.00)	14.00 (10.00, 16.00)	15.00 (11.50, 18.00)	<0.001
BMI (kg/m^2^, mean ± SD)	24.86 ± 37.72	23.36 ± 3.92	24.37 ± 19.47	23.50 ± 5.88	24.26 ± 28.47	0.153
CES-D-10 score at wave 1 (median, Q_25_–Q_75_)	4.00 (2.00, 6.00)	11.00 (8.00, 14.00)	8.00 (5.00, 12.00)	14.00 (11.00, 17.00)	7.00 (3.00, 11.00)	<0.001
CES-D-10 score at wave 2 (median, Q_25_–Q_75_)	4.00 (2.00, 6.00)	11.00 (8.00, 14.00)	8.00 (5.00, 12.00)	14.00 (11.00, 17.00)	7.00 (3.00, 11.00)	<0.001
CES-D-10 score at wave 3 (median, Q_25_–Q_75_)	4.00 (2.00, 6.00)	9.00 (6.00, 12.00)	18.00 (16.00, 21.00)	18.00 (15.00, 21.00)	6.00 (3.00, 11.00)	<0.001
Healthy lifestyle, N (%)						0.0212
0–1 healthy lifestyle	2205 (45.85)	1335 (48.39)	309 (50.16)	324 (50.47)		
2 healthy lifestyle	2604 (54.14)	1424 (51.61)	307 (49.84)	318 (49.53)		

* The *p* value was derived from an overall ANOVA.

**Table 3:** Association of HbA1c levels with trajectories of depressive symptoms scores.

	SHD Group vs. LSD Group, OR (95%CI)	*p* Value	ID Group vs. LSD Group, OR (95%CI)	*p* Value	SMD Group vs. LSD Group, OR (95%CI)	*p* Value
Overall population (N = 8826)						
HbA1c levels	1.12 (1.01–1.23)	0.0261	1.2 (1.1–1.31)	<0.001	1.07 (1.01–1.13)	0.0271
						
Middle-aged adults, age < 60 (N = 5185)						
HbA1c levels	1.17 (1.04–1.32)	0.0113	1.22 (1.09–1.38)	0.0006	1.05 (0.97–1.14)	0.2209
						
Older adults, age ≥ 60 (N = 3641)						
HbA1c levels	1.02 (0.87–1.2)	0.8157	1.18 (1.03–1.35)	0.0139	1.1 (1.01–1.2)	0.039
						
Female adults (N = 4723)						
HbA1c levels	1.11 (0.99–1.25)	0.0724	1.25 (1.13–1.39)	<0.001	1.05 (0.97–1.14)	0.237
						
Male adults (N = 4103)						
HbA1c levels	1.13 (0.95–1.35)	0.1802	1.09 (0.91–1.3)	0.3505	1.11 (1.02–1.22)	0.0166
						
Adherence to healthy lifestyle (N = 4353)						
HbA1c levels	1.00 (0.75–1.32)	0.9708	0.96 (0.71–1.31)	0.7906	1.00(0.84–1.21)	0.9825
						
Not adherence to healthy lifestyle (N = 4473)						
HbA1c levels	1.14 (1.01–1.3)	0.0402	1.22 (1.08–1.37)	0.0014	1.06 (0.97–1.15)	0.2114

Note: The model was adjusted for potential confounders, including age, sex, BMI, residential area, education level, average household income, marital status, smoking status, alcohol consumption, comorbid physical diseases, cognitive function, and healthy lifestyle factors, including sleep duration and physical activity.

**Table 4:** Bootstrap analysis of HbA1C levels and healthy lifestyle on depressive symptoms.

Pathway	β	95% CI	SE	Z	P
HbA1C levels → depressive symptoms	0.226	(0.001, 0.437)	0.113	2.004	0.045
Healthy lifestyle → depressive symptoms	−0.815	(−1.009, −0.602)	0.104	−7.805	<0.001
HbA1C levels → healthy lifestyle → depressive symptoms	−0.027	(−0.055, −0.002)	0.014	−2.033	0.042

## Data Availability

The original data presented in the study are openly available at http://charls.pku.edu.cn/en, accessed on 12 April 2024.

## Supplementary Materials

The following supporting information can be downloaded at: https://www.mdpi.com/article/10.3390/brainsci14080780/s1, Figure S1. Subgroup analysis of cross-lagged panel models applied to assess HbA1c levels and depression symptoms; Figure S2. Subgroup analysis of SEM applied to assess the mediating effect healthy life style in the association HbA1c levels have on depressve symptoms.
