# Supplementary material for: Temporal Relationship between HbA1c and Depressive Symptom Trajectories in a Longitudinal Cohort Study: The Mediating Role of Healthy Lifestyles
Source: Brain Sci. 2024 Jul 31;14(8):780. doi: 10.3390/brainsci14080780 (PMC11353008; doi:10.3390/brainsci14080780)
Supplement: Supplementary file 1 [file brainsci-14-00780-s001.zip › brainsci-3081774-supplementary.pdf]

Supplementary Materials

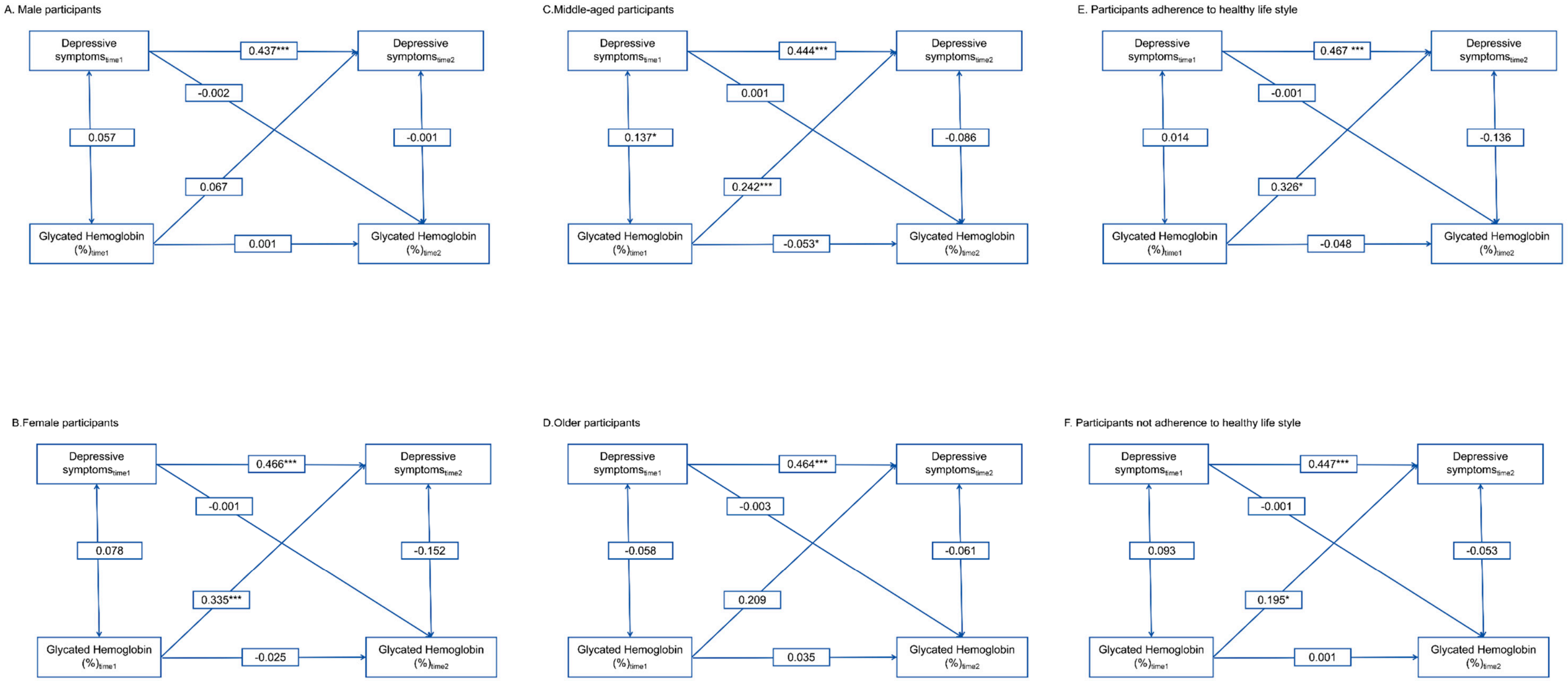

Figure S1. Subgroup analysis of cross-lagged panel models applied to assess HbA1c levels and depression symptoms. (A) in the male participants; (B) in the female participants; (C) in the middle-aged participants; (D) in the older participants. (E) in participants with healthy life style. (F) in participants with unhealthy life style.

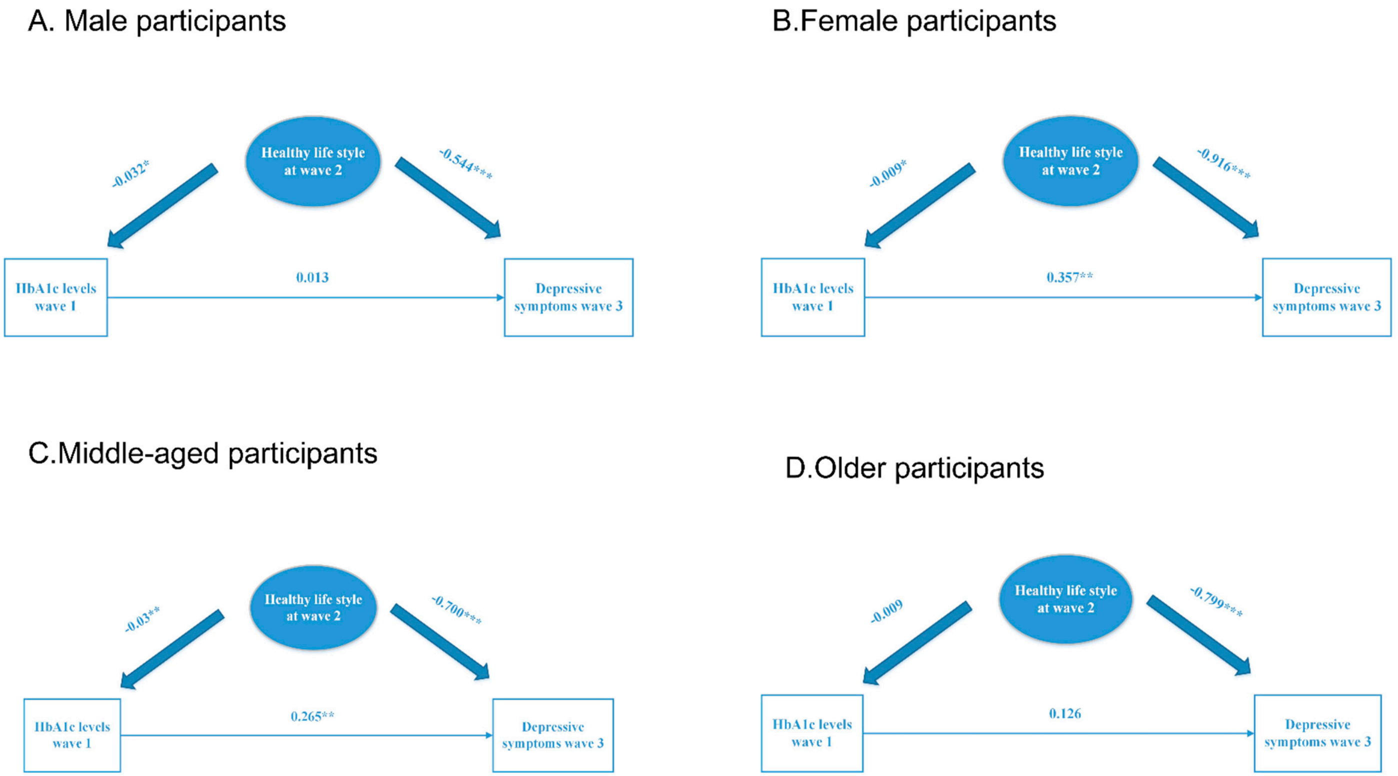

Figure S2. Subgroup analysis of SEM applied to assess the mediating effect healthy life style in the association HbA1c levels have on depressve symptoms. (A) in the male participants; (B) in the female participants; (C) in the middle-aged participants; (D) in the older participants.
